# The Use of Digital Health by South Asian Communities: Scoping Review

**DOI:** 10.2196/40425

**Published:** 2023-06-12

**Authors:** Nasser Aldosari, Saima Ahmed, Jane McDermott, Emma Stanmore

**Affiliations:** 1 School of Health Sciences University of Manchester Manchester United Kingdom; 2 The Health Academy King Abdullah Medical City Makkah Saudi Arabia

**Keywords:** South Asians, Asia, digital health, eHealth, mHealth, mobile health, aging, ethnicity, ethnic, culturally sensitive, cultural sensitivity, inequality, inequalities, disparity, disparities, physical activity, exercise, immigrant, scoping review, experience, attitude, barrier, facilitator, opinion, adoption, digital divide, accessibility, minority, mobile phone

## Abstract

**Background:**

South Asian individuals experience a higher burden of chronic diseases and limited access to health care services compared with their Caucasian peers. Digital health interventions can enhance the delivery of health care, minimize health inequities, and consequently improve health status among minority ethnic groups. However, it is unclear how South Asian people view and perceive the use of digital health technologies to support their health needs.

**Objective:**

The aim of the review is to identify South Asian individuals’ experiences and attitudes of digital health and explore the barriers and facilitators affecting their use of digital health services.

**Methods:**

The Arksey and O’Malley methodological framework was used to guide this scoping review. Five electronic databases were examined for pertinent papers, which were augmented by searching bibliographies of the retrieved papers and gray literature. A total of 1328 potentially relevant papers were retrieved from the initial search, and the supplemental search added 7 papers to the final list of potentially included papers. Each paper on the initial inclusion list was independently reviewed, leaving 15 papers to be included in the review.

**Results:**

Data were analyzed thematically leading to the development of two overarching themes: (1) barriers to uptake of digital health and (2) facilitators of use of digital health services. There was a general consensus that South Asian communities still struggle with inadequate access to digital health technologies. Some studies suggest multiple initiatives to improve accessibility and acceptability of digital health services within South Asian communities in order to mitigate health disparities and develop a more inclusive health care system. These include the development of multiple-language and culturally sensitive interventions and digital skill development sessions. Most studies were conducted in South Asian countries, focusing on measurable outcomes of digital health interventions. Few explored the experiences and views of South Asian community members residing in the West as a minority ethnic group, for example, British South Asians.

**Conclusions:**

Literature mapping proposes that South Asian people frequently struggle with a health care system that may limit their access to digital health services, and sometimes fails to consider social and cultural needs. There is growing evidence that digital health interventions have the potential to facilitate supported self-management, which is part of the plans to adopt person-centered care. These interventions are particularly important for overcoming some of the challenges, for example, time constraints, safety, and gender sensitivity, associated with the delivery of health care interventions in minority ethnic groups such as South Asians in the United Kingdom, and thus to improve minority ethnic groups’ access to health care services to support individual health needs, and consequently enhance health status.

## Introduction

The concept of enhancing health care delivery and support through electronic means (ie, digital health) is gaining popularity worldwide [1]. Indeed, the current pandemic (COVID-19) and associated surge in health care demands necessitated a greater shift from traditional health care delivery (face-to-face) to digital health care. The term “digital health” has a wide variety of definitions in different contexts, with Oh et al [2] highlighting 51 different definitions in their systematic review. However, for the purposes of this paper, “digital health” can be generally defined as the intersection of health care with information and communication technology [3]. It can also include eHealth and mobile health (mHealth). There are various forms of digital health technologies, which include the use of electronic patient records, telemedicine for remote support and monitoring of patients, and smartphone or tablet apps to arrange medical appointments or request medication prescriptions. These methods have the potential to augment and facilitate health care delivery and meet health care targets of government departments, policy makers, and health care providers; for example, to improve patient outcomes and enhance the productivity and efficiency of the health system [4,5]. Digital health technology also has the potential to assist in providing more equitable access to health services, hence, mitigating health disparities especially for those classified as clinically vulnerable and those from minority ethnic groups, such as South Asians in the United Kingdom [6].

South Asian people account for nearly 20% of the global population. The term “South Asian” conventionally refers to individuals whose ethnic roots originate in the Indian subcontinent including India, Pakistan, and Bangladesh. In the United Kingdom, the South Asian population is the largest minority ethnic group, comprising about 3 million individuals (5.3% of the total population) [7].

The literature focusing on South Asian communities suggests that they experience poorer health, a higher burden of chronic diseases, and development of cardiovascular diseases at a younger age compared with their White peers [8,9]. A systematic review concluded that South Asian populations have higher prevalence of hypertension, cardiovascular diseases, and type 2 diabetes and are also less likely to engage in physical activities and exercise [10]. Their experience may be exacerbated by the reported ethnic disparities and inadequate access to health care services [11].

As advocates of digital health claim that it could help improve the delivery of health services and reduce health inequities among underrepresented communities, it is necessary to first explore the targeted population’s views and perceptions of using digital health technologies. This is likely to help in determining the feasibility and acceptability of digital health interventions within these communities. This paper will focus on South Asian communities as one of the largest ethnic groups worldwide.

## Methods

### Study Design

The Arksey and O’Malley [12] framework for scoping reviews was used to guide this review of the current literature pertaining to South Asian individuals’ experiences and perceptions of using digital to support their health needs. Scoping reviews aim to systematically assess the breadth, depth, and characteristics of the substantive literature [13]. They attempt to provide a broad overview of the current literature; hence, a formal quality appraisal of the included studies is not always a requirement [14,15]. This allows scoping reviews to include a broader range of literature compared to systematic reviews [16]. Although formal quality assessment is not always necessary, the methodological issues within the relevant included literature are discussed.

The framework by Arksey and O’Malley [12] for scoping reviews consists of 5 phases: identification of the research question, identification of the relevant papers, development of eligibility criteria for paper selection, charting the extracted data, and reporting the findings. The framework also includes an additional, but optional, sixth phase: consulting key stakeholders, as they may offer additional resources or new thoughts that have not been discussed in the literature yet. This review included only phases 1-5, and the reporting criteria are informed by the PRISMA-ScR (Preferred Reporting Items for Systematic Reviews and Meta-Analyses extension for Scoping Reviews) checklist [17].

### Identifying Research Question

The review question was “What are the experiences and perceptions of South Asian adults of digital health interventions to support their health needs?”

### Identifying Relevant Papers

The mnemonic population, interest, and context were used to identify search terms and keywords that were relevant to the review question [18]. Textbox 1 presents the search strategy. The ASSIA, PsycINFO, MEDLINE, Embase, and PubMed databases were searched. In addition, eHealth-related websites including EU*US eHealth Work and Digital Health & Care Scotland were searched for gray literature. To expand the scope of the review, the database search was not limited by the time of publication and included papers that were published in English.

**Search strategy.**

**Inclusion criteria**

- Paper type: published and nonpublished (gray literature) papers
- Language: English
- Population: South Asian adults
- Interest: eHealth
- Context: all health care settings

**Exclusion criteria**

- Language: non-English
- Population: other Asians, other ethnicities, age<18 years
- Interest: traditional delivery of health care

### Paper Selection

**Figure 1:**
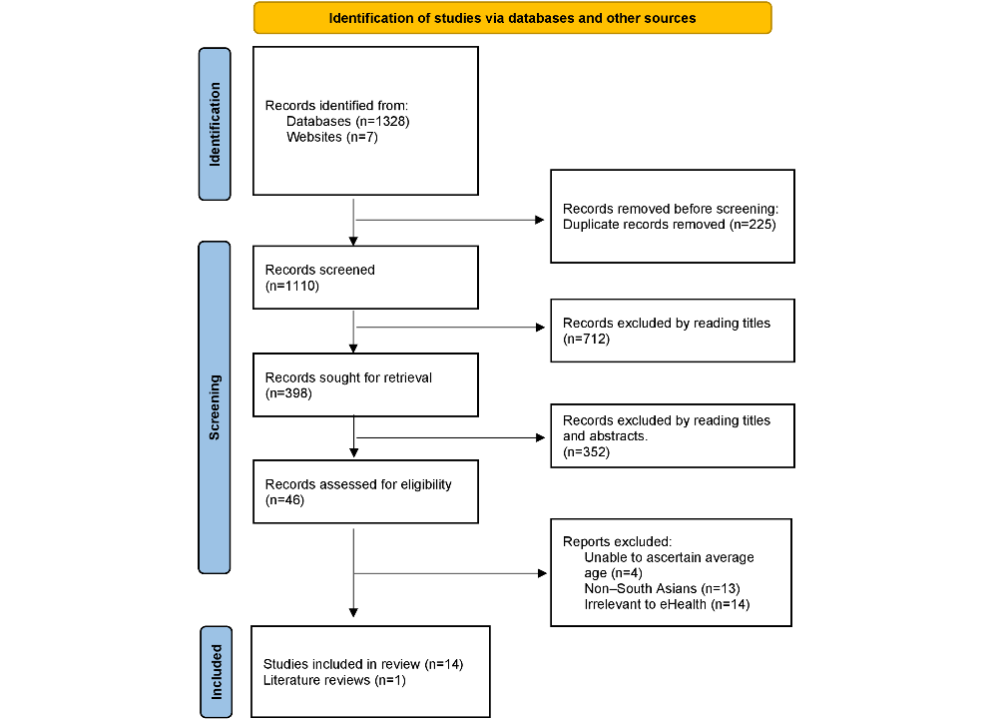
Flowchart of literature search.

The initial search retrieved 1328 papers. Seven additional papers were added from the bibliographies of retrieved papers and gray literature. Two of the authors independently reviewed the titles and abstracts of the papers potentially to be included, resulting in 46 eligible full-text papers. When the full-text papers were reviewed, a further 31 were excluded for reasons such as not being specifically related to eHealth interventions or the reviewer being unable to ascertain the ethnicity of participants, leaving 15 papers to be included in the review (Figure 1).

### Charting the Data

Relevant data were extracted from the included papers, including authors, date of publication, study sample, methods, and findings (Table 1).

**Table 1:** Charting the data.

Author (year)	Country	Study objective	Study sample	Methods	Findings
Anand et al (2016) [20]	Canada	What is the impact of messaging (emails and texting) focused on improving diet and physical activity (PA) in reducing risk for myocardial infarction (MI) among a South Asian community in Canada?	343 participants 48% females 52% males Mean age 50.6 years	Methodology: quantitative Methods: randomized control trial	The study found no difference in the change in the MI risk score after 1 year between the digital health intervention (DHI) and control groups. Despite DHI participants receiving a high number of emails or text messages compared with controls (ie, 80 vs 0) during the 12 months of the study, face-to-face contacts might have led to more behavioral change. 64.7% of participants reported they were already engaged in regular exercising, and 71.7% reported consistently following low-calorie diet. Ethnicity per se is not necessarily a barrier to use of DHIs. Heterogeneity by class, education, health beliefs, country of origin, and culture needs to be incorporated in evaluation.
DeSouza et al (2014) [21]	India	To explore the acceptability of delivering health care interventions through mobile phones among users in India	488 adults 73% females 27% males Median age 30 years	Methodology: quantitative Methods: surveys	98% of respondents preferred to receive medication adherence reminders via mobile phones. Most respondents preferred voice calls (n=419, 86%). Those who were literate in English and 40 years of age or less were more likely to use the text messaging function than those who were not. The older the participant the more preference for voice calls over texts. 4% of respondents were afraid of misuse of contact details with regard to speaking with their doctor over the phone.
Hoque et al (2017) [22]	Bangladesh	To investigate factors that influence patients to adopt and use eHealth apps in Bangladesh	318 participants 43% females 57% males Mean age 40 years A convenience sample	Methodology: quantitative Methods: surveys	The study determined that perceived ease of use and perceived usefulness and trust were significant factors influencing the intention to adopt eHealth. Gender was strongly associated with the adoption and use of eHealth services.
Horne et al (2018) [23]	United Kingdom	To identify interventions focused on increasing PA levels among South Asian adults and identify the specific changes in the content and delivery mode of these interventions	—^a^	A systematic review	Adapting content of intervention materials, images and messages to take into account language, dress seemed to improve PA uptake. A text-based messaging service was used as part of PA interventions, as reminders and encouragement. Involving South Asian community in developing culturally appropriate interventions seems to be important in their acceptability, delivery, and uptake. Community-based participation in intervention planning, evaluation, and research appears to produce culturally and linguistically tailored interventions that address core values, attitudes, beliefs, and norms, and encourage participation in PA.
Hossain et al (2019) [24]	Bangladesh	To explore the factors that influence rural end users’ acceptance of eHealth in Bangladesh	292 adults 30% females 70% males	Methodology: quantitative Methods: surveys	Age, gender, and education have a significant impact on participants’ acceptance of eHealth. Participants who have been exposed to advertisements or promotional campaigns are more likely to use eHealth. Participants having a positive attitude toward eHealth and believing in its effectiveness are more likely to use it than those having a negative attitude.
Hyman et al (2022) [25]	Canada	To understand the barriers to and facilitators of digital health tool uptake experienced by South Asian community in Canada	197 participants 52% females 42% males Mean age 65 years	Methodology: qualitative Methods: focus groups	A community-based participatory action research approach and Photovoice methods were found useful in engaging the South Asian community in Canada to use digital health. Older age, lower level of education, and lack of digital health literacy were important barriers to accessing digital health services. Factors such as social support from family or community members and positive attitudes toward technology were considered facilitators to using digital health services. South Asian people are less likely to use digital tools for their health and face significant barriers to accessing them mainly because of language or cultural factors.
Kumar et al (2019) [26]	India	To assess the acceptability of mobile phone support during care and treatment in patients with tuberculosis in South India	185 adults 38% females 62% males Mean age 35 years	Methodology: quantitative Methods: surveys	Almost all participants accepted the idea of receiving medication reminders via mobile phones. However, 74% preferred voice calls over texting. About 75% of participants preferred video-based treatment over in-person. However, only 57.6% knew how to use their mobile camera. Only 27.2% of participants had used their mobile phones to contact their health care providers. eHealth intervention should consider language preference, mode of communication, and timing of communication during development and delivery of the intervention.
Makowsky et al (2021) [27]	Canada	To explore the increase, patterns, and predictors of the use of the internet, digital devices, and apps for health purposes in South Asian Canadians	706 participants 50.4 females 49.6 males Mean age 47.1 years	Methodology: mixed methods Methods: surveys and interviews	Participants who preferred written health information in a language other than English were less interested in all modes of eHealth-based support. Interest in text message–based interventions was higher in older participants. Those who rarely or never speak English at home are more likely to cite friends as a source of health information rather than the internet. Health care providers were the most common source of health information, and only less than half of all respondents reported using the internet as a source of health information. Female internet users are more likely to be web-based health information seekers. Increasing age is a negative predictor of app use for health purposes. Language preference and age were predictors of web-based health information seeking.
Prinjha et al (2020) [28]	United Kingdom	To explore the perceptions and views of British South Asian patients with type 2 diabetes on mobile health texting to support medication adherence	67 participants 57% females 43% males	Methodology: qualitative Methods: focus groups	Participants were particularly interested in content that specifically met their information needs, for example, South Asian food. Text delivered in English was perceived to be acceptable; translation to other languages was not perceived necessary. Texting can be developed into different formats and disseminated in face-to-face groups for those who do not have access to mobile phones. Texting may also have to include culturally relevant messages sent to those who opt to receive them.
Quaosar et al (2018) [29]	Bangladesh	To identify the factors that influence the elderly’s intention to use mHealth services	245 Bangladeshi	Methodology: quantitative Methods: surveys	The study shows that performance expectancy, effort expectancy, and social influence had significant influence on intention to use mHealth services.
Ramachandran et al (2015) [30]	India	To assess willingness to receive health-related information through mobile phones (mHealth intervention)	285 participants 60% females 40% males Mean age 56 years	Methodology: quantitative Methods: surveys	60% of participants showed willingness to receive health information via mHealth; 52.6% preferring voice calls. Lower age group, male gender, having formal education and employment were associated with willingness to use mHealth services.
Teo et al (2021) [31]	Singapore	To evaluate elderly Asians’ acceptance toward digital health services amid the pandemic (COVID-19)	523 participants 171 Indians 177 Malays 175 Chinese 51.4% females 48.6% males Mean age 72 years	Methodology: quantitative Methods: phone interviews	Almost all (98.1%) participants reported no prior experience in using digital health services. About half of the participants (52%) had a positive perception that eHealth could help minimize unnecessary physical contact. 77.8% of them were uncomfortable with artificial intelligence software interpreting their medical results. Older and lower-income participants were less likely to use digital health services.
Vyas et al (2013) [32]	United States	To better understand health-related behaviors within South Asian community in the United States	709 participants 55% females 45% males Mean age 34 years	Methodology: quantitative Methods: paper and web-based surveys	The top 3 sources of health information were internet (76.9%), physician (76.1%), and family (61%). 42.8% of participants picked internet as their primary source of health information. Preference for physician over internet was associated with age (older preferred physician). Participants who were not proficient in English cited friends as their primary source of health information. Only 29% of participants involved in PA 4-6 times per week for 30 min and 28% of them rarely exercised. Older participants are more likely to engage in PA.
Yasmin et al (2020) [33]	Bangladesh	To explore the perceptions of patients with type 2 diabetes of an mHealth intervention for diabetes management	10 participants 78% females 22% males Age 50-59 years	Methodology: qualitative Methods: interviews	Most participants perceived the mHealth (voice calls) positively. Most participants were happy to pay for the mHealth service. Participants suggested including monthly discussion sessions to give them the opportunity to talk, share, and discuss about their health conditions and offer free physician consultations and laboratory tests as part of the mHealth service provision.
Zibrik et al (2015) [34]	Canada	To assess eHealth literacy and explore barriers to eHealth self-management	Surveys: 896 participants 414 Punjabis 254 Chinese Focus groups: 28 Punjabis 27 Chinese 52% females 48% males 75% aged 60+ years	Methodology: case study Methods: surveys and focus groups	Participants <60 years of age were approximately 8 times more likely to know how to use the internet to answer their questions about health than participants >80 years of age. Lower income and educational attainment were negatively associated with use of eHealth. Participants who self-reported lower educational level (elementary or none) were 5 times less likely to know what health resources are available on the internet compared with participants who self-reported college-level education. Female Punjabis were more likely to know what health resources are available on the internet and where to find helpful health resources on the internet compared with male participants.

^a^Not available.

## Results

### An Overview of the Findings

The 15 papers included emanated from different parts of the globe, most from Canada (n=4) and Bangladesh (n=4), followed by India (n=3). Other countries generated 1 or 2 papers.

Different methodologies were used; quantitative designs, including randomized controlled trials and descriptive designs, were most frequently used (n=9). The methodological designs of the included papers were quantitative (n=9), qualitative (n=4), mixed method (n=1), and systematic review (n=1).

### Thematic Analysis of the Findings

#### Overview

Thematic analysis facilitates the identification of issues that are discussed frequently in data and can be modified to be used for reviews of literature [35]. Thematic analysis of the data and discussions was performed with NVivo 12 (Lumivero, 2018). It was noticed that terms such as “eHealth,” “mHealth,” and “digital health” were used sometimes interchangeably in the reviewed papers. For clarification purpose, in this review, “digital health” is used as a generic term to refer to all health care interventions that are delivered via electronic means (Table 2).

Thematic analysis of the included papers resulted in the development of 2 major themes, each encompasses a number of subsidiary categories.

**Table 2:** eHealth term or intervention discussed by authors.

Term	Studies
eHealth: portable health clinic (PHC)	Hossain et al [24]
mHealth: interactive calls and call center	Yasmin et al [33]
mHealth: voice calls and texting	DeSouza et al [21] and Ramachandran et al [30]
Digital health: email and texting	Anand et al [20]
Health apps and web-based sources	Makowsky et al [27]
Internet for health purposes	Vyas et al [32]
Photovoice	Hyman et al [25]
mHealth: video-based calls and in-person	Kumar et al [26]
mHealth: texting	Prinjha et al [28]
Digital health service	Teo et al [31]

#### Theme 1: Barriers to the Uptake of Digital Health

Several subsidiary categories form the overarching theme that discusses barriers to the uptake of the digital health.

##### Level of Literacy

Limited English proficiency is frequently noted as a barrier to accessing digital health services for South Asian community members, particularly older individuals. In a study of the use of digital health by senior immigrants in Canada, only 25% of the Punjabi participants reported advanced English proficiency [34]. Reading and writing fluency in the English language are assumed to be a requirement for effective use of digital health services. Moreover, in a qualitative study to explore the barriers to and facilitators of accessing a digital health intervention, “Photovoice,” most of the older South Asian Canadians had struggled to use the intervention due to their limited proficiency in English [25]. The participants also cited a problem with translation of the intervention content as it was perceived as either too academic or improperly translated. Thus, digital health interventions should avoid the use of complicated medical terminology and provide health information in a simplified (ie, everyday) language to make it more accessible for all end users.

Although provision of digital health interventions in multiple languages seems beneficial, the evidence supporting this claim is inconclusive. Prinjha et al [28] stated that the written versions of some South Asian languages may be too formal and difficult for most people to understand, and some dialects do not have a written form. Indeed, many older people from the same ethnicity reported difficulty reading or writing in any language [25]. In a cross-sectional study to explore the acceptability of using mHealth to manage tuberculosis in India, Kumar et al [26] reported that more than 20% of participants (mean age 35 years) had no formal education. Therefore, the provision of multilanguage options is not necessarily effective in enhancing the use and uptake of eHealth interventions among older South Asian people. According to Prinjha et al [28], the vast majority of British South Asians understand English, and those who speak no or little English could rely on their children or grandchildren for translation. In addition, the authors suggested the provision of health information in languages other than English might not be helpful for encouraging recently immigrated South Asians to improve their English fluency.

##### Technology Literacy

In terms of technology literacy, older South Asian individuals (aged 65 years or older) commonly rated their literacy as low or even none. The difficulties associated with using technology and accessing web-based health information were frequently mentioned as a barrier to improving digital health literacy within this population [25,27]. This lack of technology literacy might contribute to exacerbating the identified digital divide within the general community and leaving the South Asian group behind in terms of research and health policy. Kumar et al [26] stated that more than 40% (64 out of 151) of their study participants in India did not know how to use the camera on their phones. However, this percentage might have been lower if the study had included South Asian immigrants in one of the developed countries as they tend to have better level of education [32].

##### Negative Preconceptions of Digital Health

Prior to introducing a new initiative, it is always important to measure the feasibility of the specific intervention and whether it is acceptable to the target community. The uptake of many digital health interventions appears to have been hampered by sociotechnical challenges, lack of trust, mismatch between design and user needs, and other adoption hurdles specific to at-risk and clinically vulnerable groups such as immigrants and older adults [23,24]. There are also concerns about the detrimental health impacts of technology (particularly cell phones) and the perception that excessive use of technology interferes with spending time with family [34]. This is an essential aspect when developing or introducing a digital health intervention, especially for people from family-oriented cultures such as South Asians [31].

##### Time Constraints

Learning to use technology, especially for health reasons, was often constrained by lack of time. Many South Asian men are involved in jobs that require long working hours and struggle with work pressure [32,33] resulting in little or no time for self-care. The social expectations of women in South Asian communities in Canada also appear to play a role in them being unable to learn or use digital health tools [25]. Moreover, due to associated familial and household duties, they are less likely to undertake the recommended amount of physical activity. However, in comparison with conventional ways of self-care such as joining a physical activity group, digital health interventions might be more accessible and supportive for South Asian people to manage and improve their health status. They (digital health interventions) could be a solution for older South Asian individuals to overcome time constraints that hinder their self-management of their health conditions.

##### Gender Sensitivity (Roles)

The roles associated with people based on their gender seem to be a factor in the likelihood of and acceptance of digital health. Makowsky et al [27] studied the use of internet and digital devices for health purposes among Canadian South Asians, suggesting that being a woman was independently correlated with lower likelihood of health-related internet use. In these communities that are largely dominated by males, females are less likely to use digital health and, in Bangladesh, they may even need permission from their male family members to buy or use a technology device [33]. This has been partly linked with socioeconomic status and social and cultural norms in South Asian communities [22,24]. In contrast, it was suggested that female Punjabi adults in Canada, compared to males, were more knowledgeable about web-based health resources and the quality of these resources [34]. This, however, could be related to the immigration status of many of these participants, who had lived in a Western country for more than 20 years, which might have altered some of their cultural norms. This is corroborated by a study of South Asian community members in Canada, which also proposed that their culture is changing toward gender equality [25].

Unlike older South Asian men, women might be more sensitive to cultural values, for example, modesty, that may limit their freedom to maintain a healthy lifestyle. In a systematic review, Horne et al [23] suggested that interventions to increase the level of physical activity that are not culturally sensitive, for example, involve exercising in a mixed gender facility, may be unacceptable or ineffective for South Asian women. Other issues that might hamper the success of these interventions include safety and unfavorable weather [33]. Thus, digital health interventions might be a suitable alternative to traditional initiatives to increase the uptake of exercise and improve health in general within South Asian communities. These digital health interventions are likely to be safer because they do not require people to leave their own home, more acceptable because they do not involve unfavorable physical mixing with the other gender, and weather-independent.

##### Aging and Self-Efficacy

The lack of confidence and fear of learning among older community members in Canada were also thought to hinder the uptake of digital health services [34]. Some older people have a misconception that they cannot learn anything new since they are 60 years or older [25]. They believe that there is too much to learn and that they are unable to absorb new concepts, which sometimes demotivates them from engaging in the learning and use of digital health services. Therefore, it is important to improve their confidence and digital literacy levels to ensure their inclusion in the new system of health care, that is, digital health. Zibrik et al [34] reported that over 65% of their South Asian Canadian participants had the desire to improve their digital skills to better manage their health conditions. This indicates that training courses to enhance older peoples’ digital literacy might be in high demand and are likely to attenuate the digital divide gap and improve their health outcomes. These courses could also help to increase the awareness of digital health interventions available. However, Hyman et al [25] suggested that the cost of training courses should be considered because some older people might struggle to afford them.

##### Lack of Trust in Digital Health Services

The literature pertinent to the use of digital health has indicated that there is an association between trust and the use of digital health services. Hoque et al [22] surveyed 326 participants in Bangladesh and argued that trust was an important prerequisite for the use of eHealth services and that there was a positive relationship between trust and intention to use eHealth. The more the patients trust digital health interventions (eg, mHealth), the more they are willing to share their health status and medical information [29]. In congruence, Hyman et al [25] reported that some older participants in Canada had relatively low trust in digital health and often opted for conventional ways, for example, visiting a doctor rather than using digital health services to seek medical advice. Moreover, those who obtain health information from the internet often feel they need to double-check with their family doctor. This preference for conventional ways could be related to the participants’ age group (>50 years), or fear of their information being misused, or it might have a cultural basis, where physical interactions are normally preferred over web-based communication.

##### Communication Preferences

Despite the recent exponential increase in the use of web-based communication, face-to-face interaction seems to remain a preference for many older South Asian individuals. Prinjha et al [28] explained that older British South Asians preferred attending face-to-face groups to discuss management of their health conditions over receiving health recommendations via texting. This was also corroborated by a study of 709 South Asian people in the United States to determine their health information resources [32]. Although the majority of participants cited the internet (42.8%), followed by physicians (36.9%) as their key resources for health information, older participants were inclined to choose physicians over the internet. The study, however, was limited to participants who were fluent in English, dismissing other South Asian adults with varying levels of English proficiency. Having conducted a randomized control trial to assess the impact of a digital health intervention (emails and texting) on improving diet and physical activity, Anand et al [20] proposed that face-to-face contacts may result in better outcomes. In contrast, the majority of patients in India (98% and 60%) preferred receiving health information via mobile phones [21,30]. However, voice calls were often preferred over texting for those participants. In the latter 2 studies, preference for texting was linked with gender, age, and literacy in English. However, the recent pandemic (COVID-19) and its consequences that have forced a shift in the health care delivery mode (from traditional to digital health) might now have changed the negative perceptions of digital health among people, especially older ones.

#### Theme 2: Facilitators of the Use of Digital Health

To overcome some of the challenges associated with successful adoption of digital health in South Asian communities, the literature has suggested several strategies such as social support and digital skills training opportunities [25,33]. The strong cultural emphasis on family life within these communities can be used to promote the uptake of digital health services. Prinjha et al [28] discussed the significant role of family members in supporting older British South Asian individuals in using technology to arrange an appointment with their doctor or request an electronic prescription. Thus, social support from family members, particularly younger adults, in South Asian culture is likely to facilitate the acceptance and use of eHealth services. Reliance on social support, however, is not necessarily a facilitator; it can sometimes be a barrier to technology use [34]. Heavy reliance of older adults on their children or grandchildren to help them with using digital health services might be counterproductive for improving the level of digital literacy among older adults. Thus, in parallel with social support, older adults should be encouraged to self-manage their health status and maintain their independence.

To enhance digital health literacy, computer classes and hands-on workshops have been cited as mitigating the digital gap between older South Asian adults and the general community in Canada [27]. These classes are likely to enable the use of health technologies, spark older people’s curiosity, and reduce their dependence on others to manage their health needs [25]. However, the site of delivery of these workshops should be carefully considered. As Horne et al [23] suggested, the use of community facilities and familiar gathering places, for example, temples, is essential for the accessibility and acceptability of community-based interventions for older South Asian people.

In terms of improving health literacy, many older adults in India suggest the use of conventional modes of communication, for example, television, radio, or even voice calls, in order to reach individuals who are less familiar with modern technology [26]. These modes can additionally be used to increase the awareness among older adults about digital health technologies and learning opportunities to improve their digital literacy. Hyman et al [25] stated that many older South Asian Canadian members, following participation in a digital health intervention (Photovoice), which promoted learning about eHealth services and health in general, highlighted the importance of the intervention being accessible and simple. This indicates that carefully designed digital health interventions that consider accessibility, ease of use, and the social and cultural preferences of the end users can effectively support older adults to self-maintain and manage their own health.

## Discussion

### Principal Findings

This scoping review sought to map the existing knowledge pertaining to the experiences and views of South Asian communities on using digital health to support their health needs. The findings indicate that South Asian populations experience limited access to digital health services. South Asian people, especially older adults, encounter multiple sociotechnical challenges that constrain their access to learning about or using digital health services, including limited English language proficiency [34], low levels of technology literacy [26,27], and lack of trust in digital health [25]. Therefore, this inadequate access to digital health services, if not addressed, may widen the digital divide gap between South Asian communities and the general population and exacerbate health disparities.

A number of authors have claimed that the development of multiple-language digital health interventions could help some South Asian community members to overcome the English language barrier [25,34]. Providing a digital health intervention in the end user’s preferred language is more likely to increase its acceptability and usability. However, it should be noted that South Asian communities are heterogeneous in terms of spoken language and that some dialects have no written form. Thus, a prior discussion with the targeted population is crucial to identify the preferred languages that should be used in the digital health intervention.

In terms of sociocultural barriers, time constraints and gender roles sensitivity have been frequently cited by older South Asian individuals, including those residing in Western countries, as major barriers to the use and uptake of digital health services [27,33]. It was reported that there is often little or no time to learn or explore digital health services due to work-related pressure or familial responsibilities. Moreover, health interventions that involve the mixing of men and women in 1 avenue (eg, gymnasium) are less preferred within South Asian communities [23]. Digital health interventions, in comparison with traditional interventions, require relatively less time as they do not require relocation (eg, going from work or home to gymnasium center) or unfavorable mixing with different genders. However, access to digital health services can also be hampered by gender inequity within South Asian countries [33]. Therefore, it is necessary to measure access to digital health tools within the targeted community prior to introducing or implementing a digital health intervention, as the level of accessibility would likely impact on the intervention usability and effectiveness.

Concerns about using digital health interventions (eg, confidentiality) and preference for conventional ways of communication are positively associated. People often opt for face-to-face health visits because they are more reliable and trustworthy compared to web-based consultation for instance. Thus, a number of studies reported that older South Asian adults prefer conventional ways of communication to meet their health needs [25,28]. However, most of these studies compared physical health visits to emails or texting, which require higher ability in reading and writing. Video calls, on the other hand, such as Teams or Zoom, can be relatively easy to use regardless of the user’s level of literacy. Therefore, older adults may find video calls easier than using emails or texting services, and equally usable compared to traditional ways, to support their health needs. However, a comparative study to compare video calls to face-to-face visits for health purposes is warranted to support this assumption.

To overcome some of the barriers to the use of digital health services, a few authors have suggested social support and digital skills training sessions for older adults [27,33]. These suggestions are expected to encourage older adults to learn and use digital health services, address their trust concerns, and improve their confidence in using new modern technology for health purposes. However, the cost of these sessions should be considered as some individuals may find it unaffordable.

Despite the South Asian communities being one of the largest ethnic groups in the world, this review identified only a relatively small number of papers that discussed their experiences and perceptions of digital health. This highlights the underrepresentation of these communities in the international literature and urges further exploration of these communities in order to improve the health status of South Asian communities and potentially mitigate current health disparities. Furthermore, there were even fewer papers that focused on South Asian people who are residing permanently in the West, for example, British South Asians. Thus, there is an increasing demand to explore the experiences and attitudes of these minority communities in order to improve their health status and ensure the development of an inclusive health care system.

### Limitations

This review of literature encountered some limitations. First, it was not clear that the findings of studies conducted in South Asian countries are transferable to South Asians residing in Western countries. Second, there was a risk of selection bias as the included papers were not formally assessed for quality. This risk, however, was reduced by the use of a dual independent review throughout the selection and data extraction processes. Moreover, the review focus was expanded from older (50+ years) South Asian individuals to include all South Asians due to the very limited number of papers retrieved by the initial search.

### Conclusions

This paper reviewed the literature on South Asian individuals’ experiences and perceptions of digital health, highlighting the barriers and facilitators affecting their use of digital health services. It has become evident that South Asian ethnic groups are generally overlooked in the international health-related literature. It is concerning that South Asian people’s health needs are not always explicitly highlighted and addressed. Yet, many health interventions have been developed and implemented aiming to improve the health status of these communities. The basis of these interventions has largely relied on international literature, which can sometimes fail to take South Asian people’s circumstances and needs into account, risking wasting their time and resources.

Digital health interventions have the potential to facilitate supported self-management, which is part of the British National Health Service’s long-term plan to adopt person-centered care. These interventions are particularly important for overcoming some of the challenges, for example, time constraints, safety, and gender sensitivity, associated with the delivery of health care interventions in minority ethnic groups such as South Asians in the United Kingdom.

## Appendix

Multimedia Appendix 1 Full details of the included papers.

## Abbreviations

mHealth: mobile health
PRISMA-ScR: Preferred Reporting Items for Systematic Reviews and Meta-Analyses extension for Scoping Reviews

## Data Availability

Full details of the included papers are submitted in Multimedia Appendix 1.
